# Identification of genes differentially expressed as result of adenovirus type 5- and adenovirus type 12-transformation

**DOI:** 10.1186/1471-2164-10-67

**Published:** 2009-02-06

**Authors:** Janet Strath, Lindsay J Georgopoulos, Paul Kellam, G Eric Blair

**Affiliations:** 1 Institute of Molecular and Cellular Biology, University of Leeds, Leeds, LS2 9JT, UK; 2 Department of Biology, University of York, Heslington, York, YO10 5DD, UK; 3 Department of Infection, Division of Infection and Immunity, University College London, London, W1T 4JI, UK

## Abstract

**Background:**

Cells transformed by human adenoviruses (Ad) exhibit differential capacities to induce tumours in immunocompetent rodents; for example, Ad12-transformed rodent cells are oncogenic whereas Ad5-transformed cells are not. The E1A gene determines oncogenic phenotype, is a transcriptional regulator and dysregulates host cell gene expression, a key factor in both cellular transformation and oncogenesis. To reveal differences in gene expression between cells transformed with oncogenic and non-oncogenic adenoviruses we have performed comparative analysis of transcript profiles with the aim of identifying candidate genes involved in the process of neoplastic transformation.

**Results:**

Analysis of microarray data revealed that a total of 232 genes were differentially expressed in Ad12 E1- or Ad5 E1-transformed BRK cells compared to untransformed baby rat kidney (BRK) cells. Gene information was available for 193 transcripts and using gene ontology (GO) classifications and literature searches it was possible to assign known or suggested functions to 166 of these identified genes. A subset of differentially-expressed genes from the microarray was further examined by real-time PCR and Western blotting using BRK cells immortalised by Ad12 E1A or Ad5 E1A in addition to Ad12 E1- or Ad5 E1-transformed BRK cells. Up-regulation of RelA and significant dysregulation of collagen type I mRNA transcripts and proteins were found in Ad-transformed cells.

**Conclusion:**

These results suggest that a complex web of cellular pathways become altered in Ad-transformed cells and that Ad E1A is sufficient for the observed dysregulation. Further work will focus on investigating which splice variant of Ad E1A is responsible for the observed dysregulation at the pathway level, and the mechanisms of E1A-mediated transcriptional regulation.

## Background

The study of human adenoviruses (Ad) has made major contributions to our understanding of gene expression, cell cycle regulation and cancer [1]. The Ad12 serotype was originally classed as 'highly' oncogenic in the newborn Syrian hamster model due to its propensity to induce local tumours within 1 to 2 months following injection by various routes [2,3]. In contrast, Ad5 does not induce tumours in newborn hamsters [4,5]. Furthermore, although all human Ad serotypes can transform primary baby rat kidney (BRK) cells *in vitro*, only BRK cells transformed by the species A human Ads (such as Ad12) can induce tumour formation in immunocompetent adult rodents [reviewed in [6]]. In addition, substantial evidence indicates that cells transformed by species A Ads actively evade the cellular immune system [6]. Investigation of the mechanisms directing such oncogenesis has bestowed a greater understanding of cell cycle control and apoptosis; for example, it is well established that products of Ad E1A and E1B genes target the retinoblastoma gene product (pRb) and p53 genes, respectively [5,6]. Binding of pRb by E1A and subsequent release of E2F plays a central role in cell cycle progression and proliferation of infected cells. In the presence of E1B 55 kDa protein expression, this deregulated cellular proliferation is enhanced due to subversion of cell cycle control exerted by p53. Furthermore E1B-19K, a bcl-2 homolog, suppresses apoptosis. Although Rb and p53 are undoubtedly critical players in cell transformation, in order to gain a better understanding of the extent of changes involved in the process of oncogenesis, the challenge is to understand the extent of dysregulation of all the cellular networks and gain a more thorough insight of how the immune response is avoided.

All human Ad serotypes can subvert the apoptotic response to the presence of foreign viral DNA within the cell, however only a small subset of Ads possess the ability to outmanoeuvre the immune surveillance system of immunocompetent rodents, giving rise to a tumour resulting from uncontrolled cell division. The only viral genes found to be necessary and sufficient for cell transformation by Ad5 are the early genes E1A and E1B. The Ad E1A proteins lack enzymatic activity and are incapable of directly binding to host cell DNA yet can dysregulate host cell gene expression by acting as transcriptional activators or repressors and can also affect the activity of proteins of the host cell [reviewed in [6]]. Adenovirus E1A has been shown to reduce levels of surface MHC class I expression on the surface of rat, human, mouse and hamster Ad12-transformed cells, compared to cells transformed with non-oncogenic Ad5 [reviewed in [5]]. However, given the complexity of interactions and pathways *in vivo*, it is unlikely that MHC class I down-regulation alone determines the oncogenicity of serotypes such as Ad12. In addition, MHC class I down-regulation does not involve a region known to be essential for tumorigenesis that consists of 20 amino acids unique to Ad12 E1A and resides between two regions conserved in both E1A sequences of the Ad12 and Ad5 serotypes [reviewed in [6]].

The persistence of cells either infected with or transformed by DNA tumour viruses is dependent not only on the avoidance of the immune system but also on dysregulation of a complex network of cellular pathways. As a first step to address this issue, we have examined the global changes to cellular gene expression that occur as a result of adenovirus transformation of primary BRK cells. Microarray studies of adenovirus-transformed cells [7,8] as well as cells infected with adenovirus [9-11] or pLPC retroviruses carrying the adenovirus 12S E1A gene [12] indicate that numerous host cell genes are targets for dysregulation. We therefore hypothesize that the comparative analysis of gene profiles from untransformed BRK cells and BRK cells transformed with Ad12 or Ad5 E1 will, to a large extent, reveal expression differences between cells transformed with oncogenic versus non-oncogenic adenovirus serotypes and provide candidate genes and pathways involved in the processes of transformation and oncogenicity.

Here, we report the gene expression profiles of normal BRK cells and BRK-derived cells transformed with the E1 region of adenovirus 12 (Ad12 E1-TC) or Ad5 (Ad5 E1-TC). Overall 232 genes were differentially expressed in normal versus Ad-transformed BRK cells. After data normalisation, exclusion of genes with less than a 1.5 average fold-change in expression and bioinformatics analysis, we were able to assign identities to 193 out of these differentially expressed transcripts. Gene ontology information was available for the majority (86%) of the identified genes and the functional annotation suggests a wide spectrum of physiological changes that are likely to correspond to differences between transformed and untransformed cells. Analysis of steady-state levels of mRNAs and proteins was performed on a subset of the differentially regulated cellular genes and possible roles for these genes in adenovirus transformation (and oncogenicity) are proposed.

## Results

### Analysis of RNA transcript levels from 15,481 genes in cell-lines transformed with Ad12 or Ad5 E1 region

The microarray study was performed in order to obtain insight into how transformation with Ad12 or Ad5 E1 region affects levels of expression of RNA in primary BRK cells. Any change from normal levels may reflect viral modification of host cell RNA transcription, processing and/or stability. In total, 15,481 rat genes were screened by GeneFilters cDNA microarray releases gf300, gf301 and gf302. Excluding control spots (total genomic DNA, tgDNA, and β-actin) the total number of genes on gf300, gf301 and gf302 was 5147, 5184 and 5150, respectively. Two copies of each rat GeneFilters cDNA microarray filter set (set A and set B; each comprised of gene filter releases gf300, gf301 and gf302) were screened in duplicate with labelled cDNA derived from independent preparations of RNA from adenovirus E1-transformed and untransformed BRK cells.

### Identification of 232 differentially expressed cellular genes

**Figure 1:**
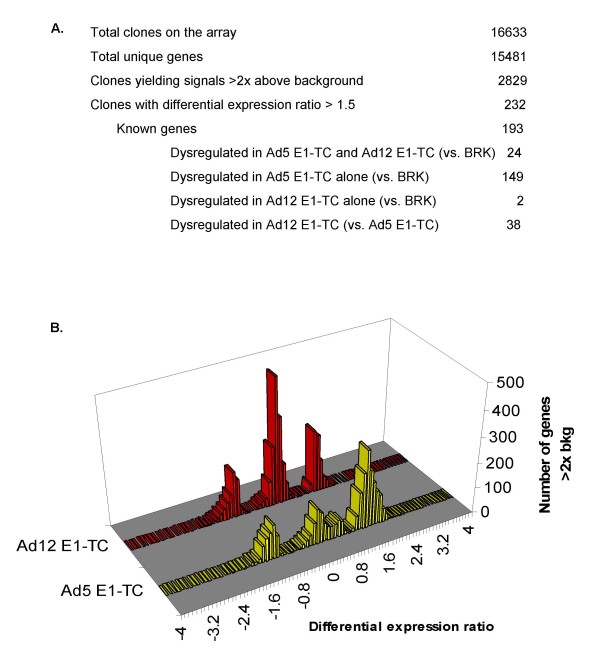
**cDNA microarray analysis of differential gene expression between non-tumorigenic Ad5 E1-TC, tumorigenic Ad12 E1-TC and untransformed BRK cells**. A. Summary of the rat GeneFilters microarray analysis. B. Distribution of differential gene expression after normalisation for all data points in Ad E1-TC relative to untransformed BRK cells. Number of genes represents those that were above twice that of the array background in at least one set of duplicate cell types.

Following normalisation, 2829 cDNAs with an expression value above twice that of background in both duplicate hybridisations of at least one cell type were selected for further analysis. Expression ratios of tgDNA between duplicate samples was consistent with good reproducibility (median tgDNA ratio: 1.16) and genes whose expression ratio was greater than ± 1.2 were investigated further. Consistent with previous studies which show cell surface expression of β2-m in Ad12-TC is reduced [13], β2-m was shown by the microarray study to be down-regulated between 1.2- and 1.34-fold in Ad12 E1-TC and is essentially unaltered in Ad5 E1-TC compared to BRK cells. However, the expression level of β2-m (clone AA818265) was below that of twice background in any of the hybridizations so was not reported in Table 1. In addition, cDNA clones for various alleles of MHC Class I heavy chain were also present on the arrays. However, although one clone (AI072399) showed upregulation of approximately 1.5-fold in Ad12 E1-TC compared to untransformed BRK cells, this was only apparent in filter set B and not filter set A so was not taken as significant; in contrast the upregulation in Ad5 E1-TC compared to untransformed BRK cells was of low variability (Table 1). Most of the cDNA clones with signals above twice that of background were below the usual 2-fold threshold used by microarray studies to define genes that are dysregulated (Figure 1). Due to the low sensitivity of the arrays, it was decided to set the threshold of fold-change to 1.5.

**Table 1:** Real-time PCR primer combinations used to amplify genes of interest.

**NCBI accession (cDNA clone)**	**NCBI accession for mRNA record**	**Primer**	**Sequence of primer**	**Position**	**Size (bp)**
AI044043	NM_001009172.2	ZBTB22 (F)	5'-GAGAGCCCGAGGCTACCCT-3'	1204 – 1222	74
		ZBTB22 (R)	5'-CCTGTTCCTCCCCCTTTTCT-3'	1278 – 1259	
AI059162	NM_022605.1	HPSE (F)	5'-GGCTCTCCACGGCTTCG-3'	270 – 286	65
		HPSE (R)	5'-GGTGCCGCCAAATCTCAA-3'	335 – 318	
AI145841	NM_199267.2	RelA (F)	5'-GGCATGCGTTTCCGTTACA-3'	152 – 170	68
		RelA (R)	5'-ATCTGTGCTTCTCTCCCCAGG-3'	220 – 200	
AI058327	XM_576071.1	MLK1 (F)	5'-ACCCCAGCTGCTACCCG-3'	359 – 375	71
		MLK1 (R)	5'-CGATGATCTCCTCCAGGGTTAG-3'	430 – 409	
AA924727	Z78279.1	COL1A1 (F)	5'-AAAACGGGAGGGCGAGTG-3'	228 – 245	80
		COL1A1 (R)	5'-GGTCCCTCGACTCCTATGACTTC-3'	308 – 286	
AI145995	AY648296.1	PPP1R1A (F)	5'-AGACTCAGGAGCAGCGTGGT-3'	559 – 578	65
		PPP1R1A (R)	5'-CCAGTGGTAGCATGTGGGC-3'	624 – 606	
AI044118	NM_001107425.1	NFAT5 (F)	5'-CCTTTAAGGATGCGCTCCAA-3'	13098 – 13117	77
		NFAT5 (R)	5'-CAGGGAGGAAAAGAAGAGAGTGAA-3'	13151 – 13174	

The NCBI accession numbers for the cDNA clones on the filters used in the initial microarray study are listed with the accession numbers for the corresponding mRNA records identified by bioinformatics analysis. Abbreviations for primers correspond to the gene symbols of the target genes; zinc finger and BTB domain containing 22 (ZBTB22), V-rel reticuloendotheliosis viral oncogene homolog A (avian) (RelA), mixed-lineage kinase 1 (MLK-1; synonym MAP3K9), collagen type 1, alpha 1 (COL1A1), protein phosphatase regulatory (inhibitor) subunit 1A (PPP1R1A) and Nuclear factor of activated T-cells 5 (NFAT5). Positions of the forward and reverse primers within the mRNA sequence are given in base pairs (bp).

### Overview of alterations in cellular gene expression induced by Ad-transformation

A conservative 1.5 fold change in gene expression between two or more cell types was used for further analysis, yielding 232 differentially expressed transcripts. Excluding expression ratios for duplicates associated with high variability (coefficient of variation of 25% and higher), the total number of RNA transcripts with known identities altered between Ad5 E1-TC and Ad12 E1-TC was 116. In comparison to untransformed BRK cells, 123 RNA transcripts of known identities were differentially expressed in the Ad E1-transformed cells with the majority of this dysregulation occurring in BRK cells stably transformed with Ad5 E1. A small subset of eleven RNA transcripts of known gene identities showed dysregulation compared to levels of expression in untransformed BRK cells as a result of the process of transformation by either Ad5 or Ad12 E1.

### Analysis of cellular functions targeted by Ad-transformation

**Figure 2:**
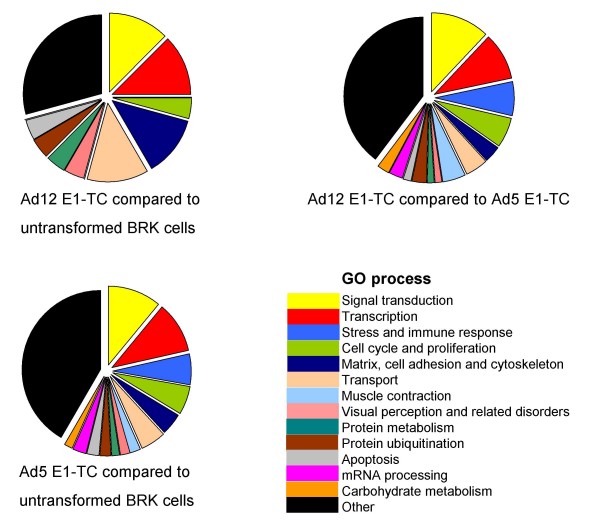
**Significantly dysregulated transcripts can be further characterised by gene ontology**. Pie charts showing gene ontology analysis of the transcripts differentially expressed as a result of Ad transformation: Ad12 E1-TC compared to untransformed BRK cell line (neoplastic transformation); Ad12 E1-TC compared to Ad5 E1-TC (oncogenic transformation compared to non-oncogenic transformation) and Ad5 E1-TC compared to untransformed BRK cell line (non-oncogenic transformation). It was possible to classify the majority of genes in to categories defined by GO biological processes with reference to the DAVID, KEGG and GenMAPP databases (see Materials and Methods). The largest category is that containing gene products that play roles in signal transduction and transcription.

The Additional file 1 summarises all of the differentially regulated transcripts, with expression levels in Ad5 and 12 E1-TC compared to each other and to untransformed BRK cells. In an attempt to identify cellular functions that are predominantly affected by Ad transformation, we searched the list of dysregulated genes for statistical over-representation of functional classes. Gene information was available for 193 clones, whereas the genes for 39 clones had not been identified (Additional file 1). Using gene ontology (GO) classifications and literature searches, known or suggested functions were used to classify 115 of the identified genes into thirteen defined categories: signal transduction; transcription; stress & immune response; cell cycle and proliferation; matrix, cell adhesion and cytoskeleton; transport; muscle contraction; visual perception and related disorders; protein metabolism; protein ubiquitination; apoptosis; mRNA processing and carbohydrate metabolism (Additional file 1 and Figure 2).

**Figure 3:**
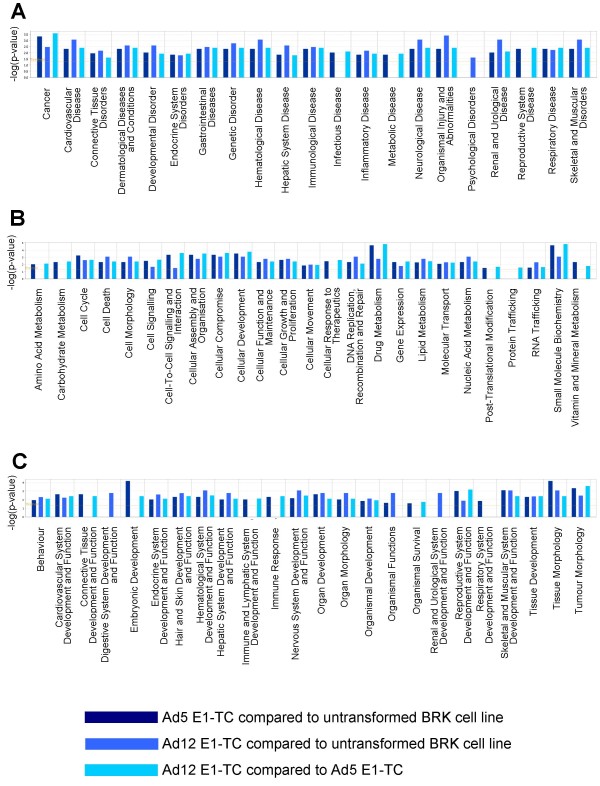
**Ingenuity comparative analysis of dysregulated targets by biofunction**. Biofunctional analysis of significant dysregulation associated with (a) disease and disorders, (b) molecular and cellular functions and (c) physiological system development and function. The significance value associated with each category is a measure of the likelihood that the association between dysregulated transcripts and a given process or pathway is due to random chance. The y-axis of each graph shows the significance, expressed as the negative exponent of the p-value calculation for each category, increasing with bar height.

**Figure 4:**
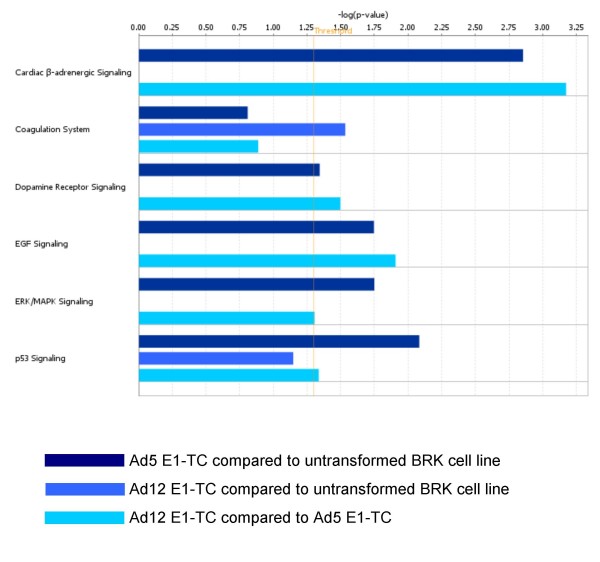
**Canonical signalling pathways affected by adenovirus transformation**. Ingenuity comparative analysis revealed several significant canonical signalling pathways affected by the dysregulation of transcripts in non-oncogenic transformation of BRK cells (Ad5 E1-TC compared to untransformed BRK cells), neoplastic transformation of BRK cells (Ad12 E1-TC compared to untransformed BRK cells) and neoplastic compared to non-oncogenic transformation (Ad12 E1-TC compared to Ad5 E1-TC).

To identify signalling pathways involved in adenovirus transformation, data sets comprised of gene expression ratios were analysed by Ingenuity. The Ingenuity Pathways Analysis (IPA) is a system that transforms large data sets into a group of relevant networks containing direct and indirect relationships between genes based on known interactions in the literature. It was possible to map 111 out of the 232 dysregulated targets with certainty to the IPA knowledge base: 66 of the mapped genes were eligible for network analysis and 51 were eligible for functions and pathways analysis. Genetic networks were created by the IPA network generation algorithm from lists of fold-change expression in Ad5 E1-TC compared to untransformed BRK cells, Ad12 E1-TC compared to untransformed BRK cells and Ad12 E1-TC compared to Ad5 E1-TC. Comparative analysis across all results was also performed and results were analysed in terms of biofunction (Figure 3) and canonical pathway (Figure 4).

**Figure 5:**
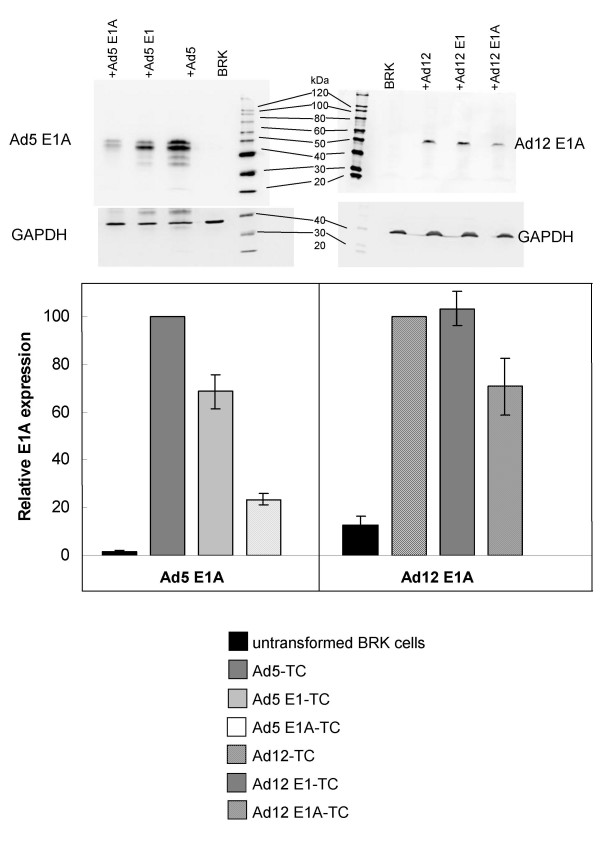
**Relative levels of E1A expression in transformed cell lines**. Levels of E1A expression in the transformed cell lines were assessed by Western Blot. Intensities were corrected for average background and normalised to GAPDH expression calculated individually for each loaded sample. The expression levels are given as percentages of the intensity for cells transformed with Ad5 or Ad12 inactivated virus. Results for Ad5 E1A expression are from an average of six membranes and values for Ad12 E1A expression represent the average from seven membranes.

From this analysis, six genes indicative of defined biological processes were selected for further study at the mRNA level by real-time PCR; V-rel reticuloendotheliosis viral oncogene homolog A (avian) (RelA), zinc finger and BTB domain containing 22 (ZBTB22), nuclear factor of activated T-cells (NFAT5), collagen type 1 alpha 1 (COL1A1), mixed-lineage kinase 1 (MLK1; synonym MAP3K9) and protein phosphatase regulatory (inhibitor) subunit 1A (PPP1R1A). The expression levels of these mRNA transcripts were investigated in the Ad E1-TCs used for the microarray study and also in BRK cells immortalised with Ad12 (Ad12 E1A-TC) or Ad5 E1A (Ad5 E1A-TC) alone. Expression of COL1A1 was investigated at the protein level by immunofluorescence microscopy and expression of RelA, MLK1 and PPP1R1A was also investigated at the protein level using cell lysates derived from the previously mentioned cell-lines and also from cells transformed with inactivated Ad5 (Ad5-TC) or inactivated Ad12 (Ad12-TC). Relative levels of E1A expression in these cell lines are indicated in figure 5. Differential splicing of the E1A transcript and phosphorylation of the E1A protein gives rise to several bands ranging from approximately 35 to 47 kDa [14]. The cell line transformed with inactivated Ad5 showed the highest level of E1A expression in comparison with the cell lines transformed with Ad5 E1 or E1A alone. This could be related to the number of copies of the viral DNA sequences that have been integrated in each cell line. Previous studies have shown that adenovirus DNA integrates at multiple sites (from around 5 to 30 copies of the viral genome per cell) and these are subject to methylation and loss/rearrangement, such that only the transforming (E1A and E1B) are constitutively expressed in the transformed cell thus driving cell proliferation [15]. Expression of E1A in cell lines immortalised by the AccI-H fragment corresponding to the 4.7% of the Ad12 genome (Ad12 E1A-TC) or the HpaI-E fragment corresponding to the left-terminal 4.5% of the Ad5 genome (Ad5 E1A-TC) is lower than that shown by cell lines transformed by the entire E1 region, in agreement with previous observations that the E1B region acts to increase the frequency and stability of transformation by E1A [16]. The lowest levels of E1A expression are in the Ad5 E1A immortalised cell-lines; probably due to the absence of the poly (A) signal of E1A in the HpaI-E fragment of the Ad5 genome used to immortalise this cell line [17].

### Genes dysregulated in Ad5 E1-TC relative to untransformed BRK cells

**Figure 6:**
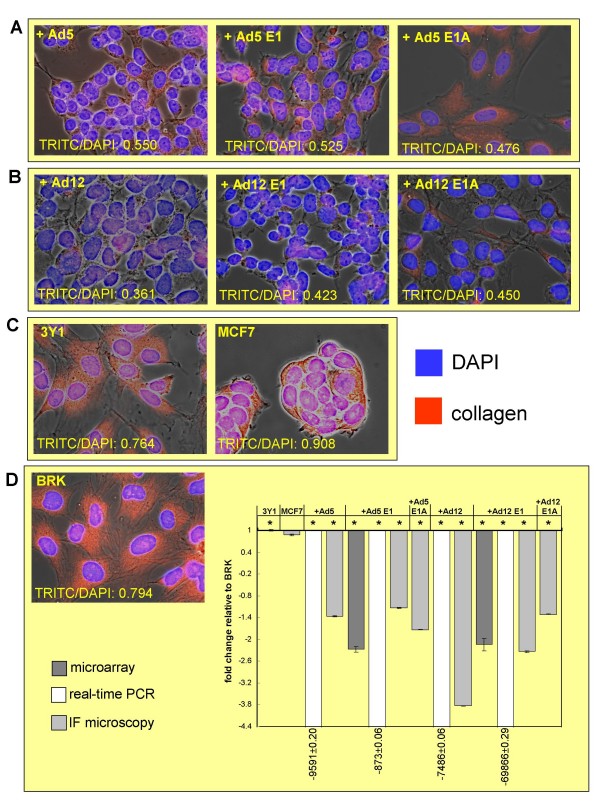
**Expression of collagen type I observed using immunofluorescence microscopy**. Immunofluorescence on cells stained for collagen type I (red) and nuclei staining with DAPI (blue) shows down-regulation of collagen as a result of transformation of BRK cells by Ad5 E1A (Panel A) or Ad12 E1A (Panel B) in comparison to untransformed rat fibroblast cell line 3Y1 and human breast tumour derived cell line MCF7 (Panel C). Representative images of one field are shown with corresponding TRITC:DAPI ratio for the individual field. Expression levels were calculated relative to intensity in untransformed BRK cells (Panel D). The dark grey bars represent the microarray results and the white bars represent the real-time PCR results while the light grey bars represent the immunofluorescence microscopy results. Each bar represents average ratio of intensity (corrected for background) of TRITC:DAPI for 10 fields imaged from same slide with significant results indicated by * (*P *< 0.500). Expression of collagen type I in MCF7 cells has been shown to vary according to the source of the cell line [86] and our results indicate variance on an individual cell basis (*P *0.860).

Functional analysis of the Ad5 E1-TC vs BRK dataset showed that the most significant disease which was associated with the mapped genes was cancer, consistent with the transformed phenotype. Eleven genes from the submitted expression dataset (CD3G, HPSE, TFAP2A, COL1A1, STMN1, CD19, GADD45B, MTA3, TFPI, ATM, EGFR) met the threshold p-value of 0.05 (actual p-values range from 4.64 × 10^-4 ^to 3.30 to 10^-2^), so are classified in this high-level category by IPA (Figure 3a). Our results agree with previous microarray results indicating up-regulation of GADD45B as a result of adenovirus 2 infection of HeLa cells [9]. In addition, in concordance with previously findings that suggest Ad5 E1 induces down-regulation of both EGFR [18,19] and COL1A1 [20], our microarray results showed down-regulation of both EGFR (2-fold) and COL1A1 (over 2-fold) in Ad5 E1-TC compared to untransformed BRK cells (Additional file 1). COL1A1 is the most highly differentially expressed target involved in cellular adhesion as identified by the microarray study and was investigated further. Significant down-regulation of COL1A1 in both Ad12 E1-TC and Ad5 E1-TC compared to untransformed BRK cells was observed at the RNA level by real-time PCR and at the protein level by immunofluorescent antibody staining (Figure 6).

**Figure 7:**
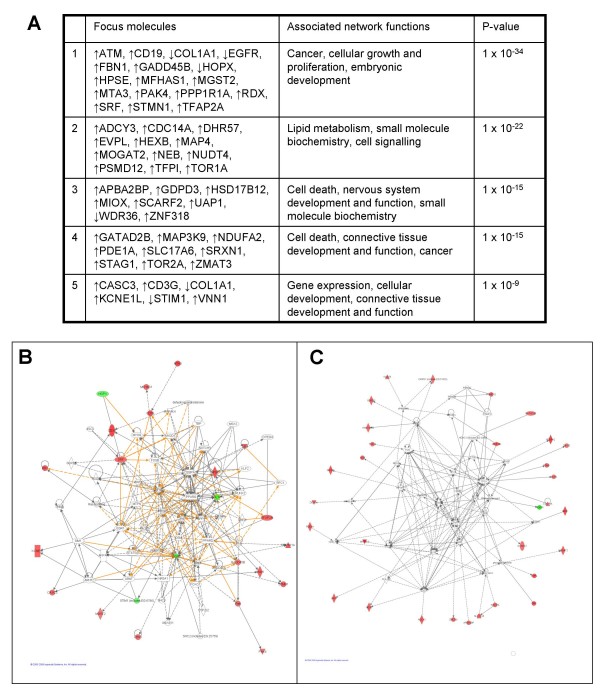
**Ingenuity network analysis of the dysregulation in Ad5 E1-TC compared to untransformed BRK cells**. The highest scoring biofunctions associated with the five top scoring networks are summarised (a) with the significantly dysregulated transcripts (focus molecules) that mapped to each network listed. Significant overlap between networks was calculated for (b) networks 1 and 5, and (c) networks 2, 3 and 4: focus molecules are depicted as down-regulated (green) or up-regulated (red).

A summary of the five top scoring networks generated by IPA based on the fold-change in gene expression in Ad5 E1-TC compared to BRK cells shows that EGFR and COL1A1 are part of the highest scoring network of genes (Figure 7a, b). The most significant function for this network is cancer, with twelve targets dysregulated over 1.5-fold (ATM, CD19, EGFR, MTA3, PAK4, SRF, STMN1, TFAP2A, HPSE, GADD45B, RDX, and COL1A1) mapped to this network being classified in this disease category with p-values ranging from 5.31 × 10^-6 ^to 1.43 × 10^-2^. The process associated with cancer that is most significantly affected by the dysregulated genes in this network is the category of developmental process of tumour cell lines (p-value 5.31 × 10^-6^). This category encompasses an assortment of findings involving the effect of dysregulation on the growth and differentiation of tumour cells: ATM protein increases cellular growth [21] and SRF and TFAP2A increase differentiation [22,23], whereas up-regulation of CD19 and down-regulation of EGFR are associated with a decrease in cellular growth [24,25].

**Figure 8:**
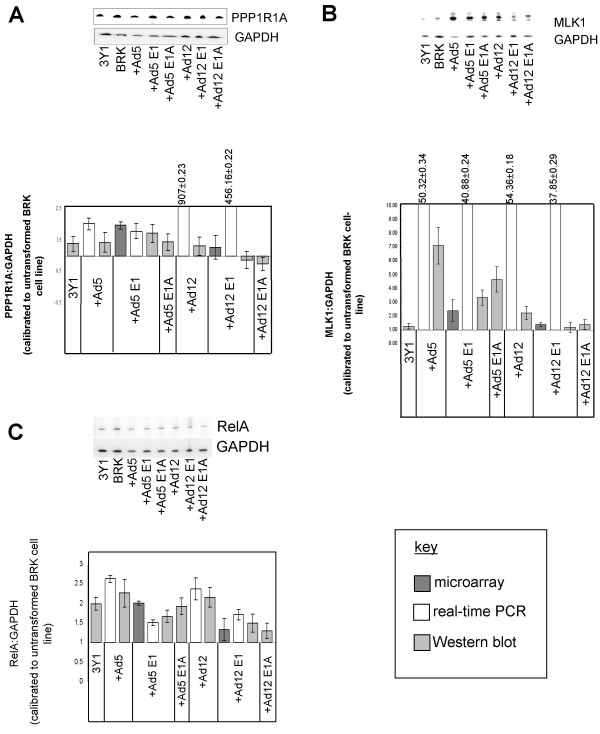
**Biological validation of microarray results by real-time PCR and Western Blotting**. Expression levels of (a) protein phosphatase 1, regulatory (inhibitor) subunit 1A (PPP1R1A), (b) mixed lineage kinase 1 (MLK1, synonym MAP3K9) and (c) RelA were investigated at the RNA level by microarray analysis (dark grey bars) and real-time PCR (white bars) and at the protein level by Western Blotting (light grey bars). Examples of membranes stained for the target protein of interest then subsequently GAPDH are given. The average intensity ratio of target protein of interest to GAPDH (calibrated to that in untransformed BRK cells) was calculated using density of band signals. The microarray results were obtained from two copies of each membrane screened in duplicate. The real-time PCR results represent three RNA samples per cell-line run in triplicate.

PPP1R1A is also present in the highest scoring network (Figure 7b). PPP1R1A inhibits PP1, a major serine/threonine protein phosphatase that plays roles in diverse pathways, namely carbohydrate metabolism, cell cycle, protein synthesis, muscle contraction and neuronal signalling. Protein phosphatase inhibitor 1 mRNA is widely expressed in mammalian tissue and, when phosphorylated by cAMP-dependent protein kinase (PKA) on Thr-35, the protein product PPP1R1A (also known as inhibitor-1) is a potent and specific protein phosphatase 1 (PP1) inhibitor [26]. PPP1R1A is closely related in terms of both structure and function to the more extensively investigated dopamine and cyclic AMP regulated phosphoprotein of relative molecular mass 32,000 (DARPP-32), which is predominantly found in the cytoplasm of neurons of the neostriatum [26]. The microarray study suggested that levels of PPP1R1A were increased nearly 2-fold in Ad5 E1-transformed cells over untransformed BRK cells, which was confirmed by the real-time PCR data. Up-regulation of PPP1R1A at the protein level in Ad5-TC, Ad5 E1-TC and Ad5 E1A-TC was also observed (Figure 8a).

Many of the pathways in the merged networks shown in Figure 7c converge on FOS, one of the downstream effectors of the MAPK/Erk signalling cascade involved in growth and differentiation, which the comparative analysis suggests is significantly dysregulated in the Ad5 E1-TC (Figure 4). Expression of c-fos is suppressed in some human tumours [27] and also in rat embryo fibroblast cells transiently transfected or stably transformed by Ad E1A and Ras, acting on chromatin remodelling factors [28]. The extracellular signal-regulated kinase (ERK) pathway is one of three major mammalian mitogen-activated protein kinase (MAPK) signalling pathways; the others being the p38 MAPK pathway, and the c-Jun NH_2_-terminal kinase (JNK) pathway.

A component of the JNK pathway, MAP3K9, was classed as the highest scoring mapped molecule by IPA assessment of fold-change regulation in Ad5 E1-TC compared to untransformed BRK cells and plays a role in the merged high scoring networks (Figure 7c). MAP3K9 (synonym MLK1) is a member of the MLK family of serine/threonine kinases and is known to activate the JNK pathway by phosphorylating (activating) MKK7 (symbol MAP2K7) upstream of JNK [29]. MLK1 is thought to be activated by the small GTP binding proteins Rac1 or Cdc42. As Rac1 or Cdc42 are known to activate both the JNK and the p38 pathways, it has been suggested that MLK1, like MLK3, is capable of activating both the JNK and the p38 pathway by activating MKK4. Several lines of evidence suggest that over-expression of c-Jun by the JNK pathway plays an essential role in transformation and tumorigenesis [30]. Furthermore, MLK1 has been found expressed at the mRNA level in epithelial tumour cell lines of colonic, breast and esophageal origin [31].

The microarray data suggested that MLK1 is up-regulated in Ad5 E1-transformed cells compared to untransformed BRK cells by 2.39-fold (Additional file 1) which was confirmed by real-time PCR (Figure 8b). Significant up-regulation of MLK1 at the protein level was observed in Ad5-TC, Ad5 E1-TC and Ad5 E1A-TC. The elevated expression of MLK1 in adenovirus-transformed cells in comparison to BRK cells could be contributing to activation of the JNK pathway leading to the transformed phenotype.

### Genes dysregulated in Ad12-TC relative to untransformed BRK cells

**Figure 9:**
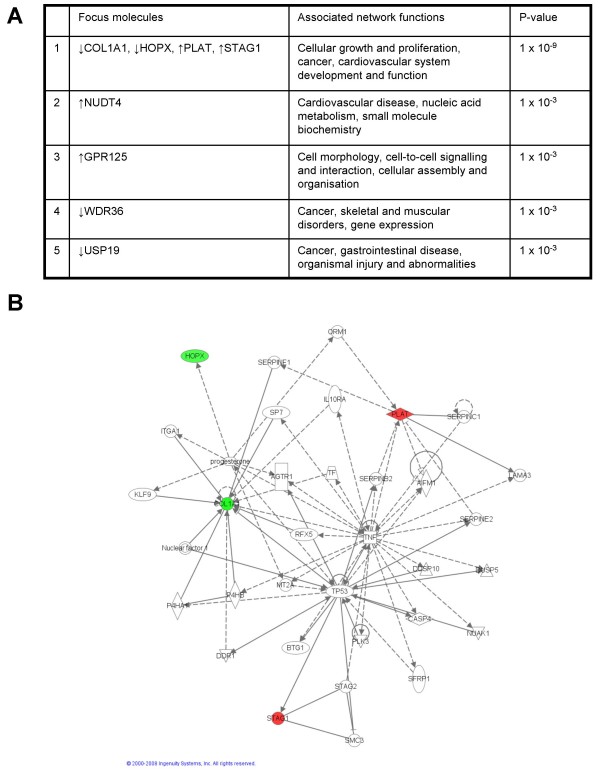
**Ingenuity network analysis of the dysregulation in Ad12 E1-TC compared to untransformed BRK cells**. Overall, less dysregulation of transcripts in Ad12 E1-TC compared to untransformed BRK cells was found by the microarray study so not many focus molecules were returned by IPA (a). The top scoring network (b) contains two molecules associated with cancer; HOPX and PLAT.

Notably fewer genes were found to be dysregulated by over 1.5 fold by Ad12 E1 transformation of BRK cells compared to Ad5 E1 transformation (Figure 1) and none of the networks returned by IPA overlap (Figure 9a). The top scoring network (p-value 1 × 10^-9^: figure 9b) generated by IPA analysis of genes dysregulated in Ad12 E1-TC compared to untransformed BRK cells only contains two mapped molecules dysregulated over 1.5-fold (COL1A1 and PLAT) that are classified as associated with cancer.

Both COL1A1 and PLAT have been found to increase the proliferation of tumour cells [32,33]. Up-regulation of COL1A1 is involved in progression of gastric carcinoma [34] and the methylation status of the COL1A1 promoter is inversely proportional to COL1A1 expression in human cancer cell lines [35], so it is possible that dysregulation of COL1A1 is an effect of the chromatin remodelling capability of E1A. Down-regulation of COL1A1 protein was observed in both Ad5 E1-TC and Ad12 E1-TC compared to untransformed BRK cells (Figure 6). A difference between the invasive properties of Ad12- compared to Ad5-TC would be expected as Ad12-TC can form tumours in both immunocompetent and immunodeficient mice. Ad12-TC cells are known to prefer attachment to collagen type IV over collagen type I [36], but to what extent this affects the metastasis of these cells is unknown. Interestingly, collagen type IV expression is affected by one of the targets dysregulated uniquely in Ad12-TC cells; tissue-type plasminogen activator (PLAT), a secreted serine protease which plays a pivotal role in ECM production and degradation. The main role of PLAT is to convert the proenzyme plasminogen to plasmin. Plasmin directly degrades components of the ECM and amplifies this effect by activating matrix metalloproteinases (MMPs) [37,38]. Given the importance of PLAT for ECM homeostasis, presumably the up-regulation of PLAT mRNA, which is associated with human pancreatic adenocarcinoma [39] and colorectal cancer [40] contributes to tumour metastasis. PLAT was found to be up-regulated over 1.5-fold by Ad12 E1 transformation but not significantly dysregulated by Ad5 E1 transformation (Additional file 1).

The two other genes in the highest scoring network that were dysregulated in Ad12 E1-TC compared to untransformed BRK cells were stromal antigen 1 (STAG1: a component of the p53 signalling pathway that plays a role in the cell cycle as it encodes a component of cohesion) and Hop homeobox (HOPX). Studies in transgenic mice suggest that, although incapable of directly binding to DNA, HOPX acts as a repressor of serum response factor (SRF) dependent transcription by recruiting histone deacetylase (HDAC) activity [41]. The loss of human HOPX (synonyms NECC1 and LAGY) expression evident in choriocarcinoma is thought to be involved in malignant conversion of placental trophoblasts and has lead to the suggestion that HOPX is a candidate tumour suppressor gene [42]. Furthermore, a link between human HOPX and lung cancer has also been proposed, based on the observation that this gene was significantly down-regulated in primary lung tumours compared to normal lung tissue samples [43]. Interestingly, uterine expression of HOPX in the progesterone receptor knockout (PRKO) mouse has been found to be increased by injection of progesterone [44]; a steroid hormone known to decrease the risk of developing uterine cancer.

### Genes dysregulated in Ad12-TC relative to Ad5-TC

As transformation by Ad12 E1 is highly oncogenic compared to transformation exerted by the nonocogenic serotype, investigation of differences in gene expression between Ad12-TC and Ad5-TC should provide a foundation that allows mapping and identification of genes involved in neoplastic transformation. This section provides insight into the comparative expression of genes in Ad12 E1-TC compared to Ad5 E1-TC in terms of networks. However, it is important to note that any difference between the response to Ad12 E1 and Ad5 E1-TC *in vivo *will involve additional factors; for example, factors involved in the immune response of the recipient animal to transformed cells are not considered here.

**Figure 10:**
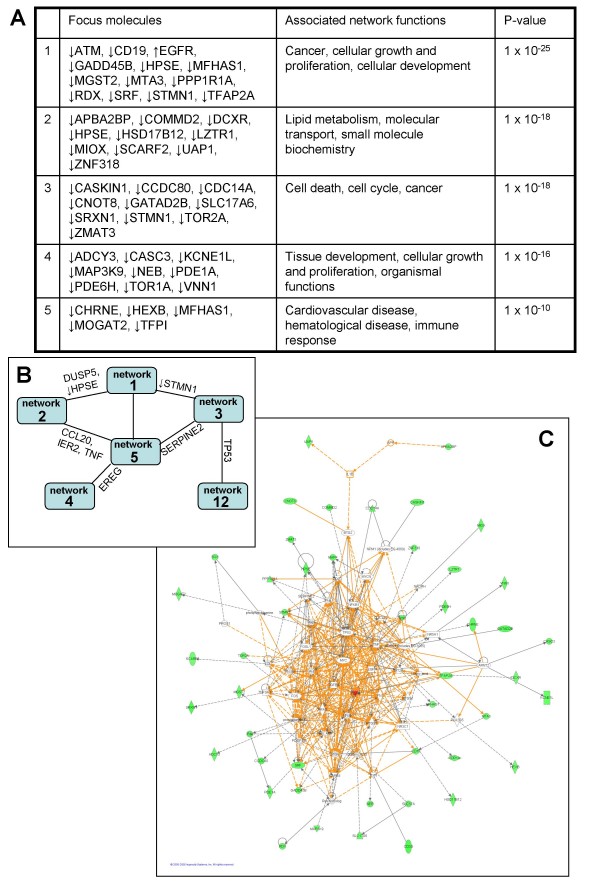
**Ingenuity network analysis of the dysregulation in Ad12 E1-TC compared to Ad5 E1-TC**. The top five highest scoring networks are summarised (a) with the most significant associated network functions and focus molecules, and (b) there was considerable overlap between networks (shared molecules are indicated). The simplified merged network (c) gives an idea of the complicated cross-talk between networks 1, 2, 3, 4, 5 and 12, with molecules identified as down-regulated or up-regulated by the microarray study depicted in green or red, respectively.

There is considerable overlap between the top five networks that scored highest by IPA (Figure 10), with the majority of molecules in the merged network associated with cancer (*p*-value range of 3.98 × 10^-15 ^to 1.85 × 10^-6^). A total of 56 molecules are annotated as affecting neoplasia and eight of these are dysregulated over 1.5 fold in Ad12-TC compared to Ad5-TC: down-regulation of ATM, CD19, CD3G, GADD45B, HPSE, TFAP2A and TFPI was observed, whereas levels of EGFR were found to be up-regulated (Additional file 1).

The microarray results also suggested significant down-regulation of two components of the p53 signalling pathway in Ad12-TC compared to Ad5-TC; ATM and GADD45B. Expression of these targets was not significantly dysregulated in Ad12-TC compared to BRK cells, yet was significantly up-regulated in Ad5-TC compared to BRK cells. Therefore, it could be possible that either this represents a unique effect of product(s) from the Ad5 E1 region on the p53-signalling pathway or that a unique Ad12 E1 gene product is negating the effects of a gene product common to both Ad5 and Ad12 E1 regions. The role of ATM in neoplasia is not due to dysregulated levels of expression: mutated ATM plays a role in neoplasia [45] and the mouse ATM gene decreases the development of neoplasia by acting through V(D)J recombination [46].

Down-regulation of GADD45B has been associated with neoplasia [47]; however levels of GADD45B were not significantly dysregulated in the BRK cell line stably transformed by oncogenic Ad12 E1 in comparison to untransformed BRK. The down-regulation of GADD45B in Ad12 E1-TC compared to Ad5 E1-TC is due to significant up-regulation of GADD45B by Ad5 E1 transformation, as previously discussed. Another dysregulated target affected by p53 is TP53I13 (synonym DSCP1); a p53-inducible gene with no GO annotation available (Additional file 1). Levels of TP53I13 were up-regulated over 1.5-fold in Ad5 E1-TC over Ad12 E1-TC and could be exerting a tumour suppressor effect in these cells, as there is *in vitro *evidence that such up-regulation retards cancer cell growth [48].

Another dysregulated target with no GO process available is the family with sequence similarity 84, member B gene (FAM84B; synonyms NSE2; BCMP101). FAM84B, usually involved in formation of the DNA repair complex, is up-regulated in breast cancer tissue, in which it localises to areas of cell-to-cell contact where it interacts with and may block the tumour suppressor function of the cell adhesion protein α1-catenin [49]. Up-regulation of FAM84B has also been reported in esophageal squamous cell carcinomas [50].

The microarray results suggested over 1.5-fold down-regulation of TFAP2A in Ad12-TC compared to Ad5-TC. Transcription factor activator protein-2 alpha has been suggested as a candidate tumour suppressor protein, as loss of TFAP2A protein in several human cancers is associated with enhanced tumorigenicity [51]. Loss of function of TFAP2A in cancer cells could be due to hypermethylation of the CpG island from the human TFAP2A, which strongly correlates with decreased expression of the gene in neoplastic breast epithelial cells [51].

Not all of the targets listed in Table 1 were mapped by IPA. One target of particular interest that was not mapped by IPA was the microarray clone AI145841, identified as Rattus norvegicus v-rel reticuloendotheliosis viral oncogene homolog A (avian) by manual BLAST searching of the non-redundant nucleotide database (100% query coverage, E-value: 3 × 10^-70^). RelA is a component of the NF-κB complex and is involved in transcriptional regulation. NFκB1 binds to RelA to form the p50 (NFκB1)/p65(RelA) heterodimer, which activates transcription of many genes in multiple tissues once phosphorylated on p65 by protein kinase A [52]. As approximately equal amounts of p65 and p50 have been previously reported to exist within the nuclei of Ad12 and Ad5-transformed cells [53,54], it would be reasonable to assume that the expression of RelA would not vary greatly as a result of Ad12-transformation. However, others have reported that Ad12 E1A 13S can block the proteolytic processing of the inhibitor of NFκB (p105) resulting in unusually high levels of the inhibitor within host cells that sequester NFκB to the cytoplasm [55]. In the cell lines transformed with either the early region of Ad12 (Ad12 E1-TC) or Ad5 (Ad5 E1-TC), as well as in cell lines transformed with either inactivated Ad12 or Ad5 (Ad12-TC and Ad5-TC), levels of RelA transcripts were slightly up-regulated compared to expression in untransformed BRK cells (Figure 8c). At the protein level, the up-regulation of RelA was least pronounced in Ad12 E1A-TC (Figure 8c). If this was to occur in infected cells, Ad12 E1A could be acting to reduce the level of RelA induction which would complement inhibition of the tumour necrosis factor receptor 1 (TNFR1) signalling pathway; such a block in proinflammatory or pro-apoptotic signalling would be beneficial for viral persistence.

**Figure 11:**
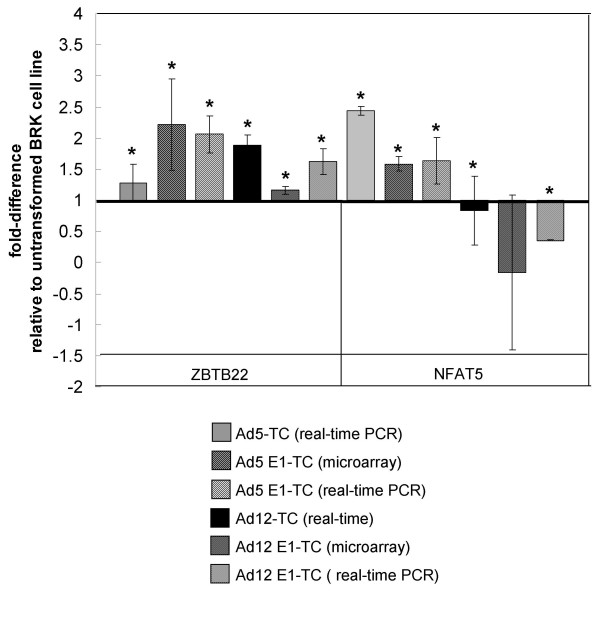
**Microarray results and corresponding RT-PCR results for ZBTB22 and NFAT5 expression in Ad-transformed cells**. The microarray results for ZBTB22 indicating up-regulation of the transcript in Ad12 E1-TC were validated by real-time PCR. The microarray result for NFAT5 indicating significant up-regulation in Ad5 E1-TC (1.58-fold; table 1) was validated by real-time PCR. The down-regulation in Ad12 E1-TC indicated by the microarray study was not significant (0.17-fold; table 1) due to high variability (p-value; 0.635) and the real-time PCR results for expression of NFAT5 also did not indicate any significant dysregulation of this transcript in Ad12 E1-TC, or Ad12-TC.

Other targets that were not mapped by IPA but were identified by manual BLAST searching include ZBTB22, NFAT5 and SSG1. No GO information about biological function is available for ZBTB22 (synonyms ZNF297 and BING1), originally identified as a gene within the 70 kbp segment flanking the TAPASIN locus on human chromosome 6 [56], within the extended collection of genes known as the Major Histocompatibility Complex (MHC). As ZBTB22 contains both a zinc finger domain and a POZ domain, the suspected function is transcriptional repression. Figure 11 shows that there is good agreement between the microarray and real-time PCR results, with up-regulation of ZBTB22 in Ad5 E1-TC and, to a lesser extent, in Ad12 E1-TC compared to untransformed BRK cells. All of the genes assigned to the stress and immune response category were found to be up-regulated in Ad5 E1A-TC compared to either untransformed BRK cells or Ad12 E1A-TC (Figure 2). These genes include NFAT5, which was confirmed by real-time PCR results as being up-regulated in Ad5 E1A-immortalised cells compared to BRK cells (Figure 11).

Steroid sensitive gene 1 (SSG1; synonyms URB, CL2, DRO1) was originally identified as an estrogen-regulated gene whose over-expression correlated with rat mammary carcinogenesis [57]. Subsequently, elevated expression of URB was reported in the adipose tissue of a strain of mice exhibiting mild late-onset obesity [58]. Our microarray results indicated significant up-regulation of SSG1 expression of over 1.5-fold in Ad5 E1-TC compared to Ad12 E1-TC (Additional file 1). Given that Ad5 E1A has previously associated with induction of CL2 in rat thyroid cells and CL2 is down-regulated in human thyroid neoplastic cell lines and tissues [59], it is tempting to consider this gene as an important player in the process of neoplastic transformation. The finding that DRO1 expression is down-regulated following neoplastic transformation of an E1A-immortalised rat kidney epithelial cell line (RK3E cells) mediated by β-catenin provides further evidence for the potential role of SSG1 in neoplastic progression [60].

## Discussion

From the information obtained about the dysregulated genes and the levels of expression determined by the microarray study, a subset of six transcripts were selected for further study at the mRNA level by real-time PCR on the basis of involvement in the immune system and/or possible involvement in oncogenesis. Expression levels of mRNA transcripts were investigated in the Ad E1-TCs used for the microarray study and also in BRK cells immortalised with inactivated Ad12 (Ad12-TC) or Ad5 (Ad5-TC) or Ad12 E1A (Ad12 E1A-TC) or Ad5 E1A (Ad5 E1A-TC). Both these results and those from studies at the protein level showed good agreement with the results obtained by microarray.

Gene ontology analysis of the microarray results revealed that the majority of classifiable dysregulated transcripts were products from genes that play roles in signal transduction and transcription (Figure 2). This is perhaps not surprising as adenovirus is known to exert a pleiotropic effect on cellular signalling pathways and perturb host cell gene expression at the transcriptional level [reviewed in [5]]. Our real-time PCR and Western blotting results confirmed dysregulation of PPP1R1A and MLK1, which play roles in signal transduction pathways, and ZBTB22 and RelA, involved in transcription processes.

The real-time PCR results did suggest highly increased expression of PPP1R1A as a result of transformation with inactivated Ad12 or the Ad12 E1 region alone; however, this contradicts both the microarray and WB results, which indicate no significant dysregulation in Ad12-TC, Ad12 E1-TC or Ad12 E1A-TC. Significant up-regulation of PPP1R1A was observed in Ad5-TC, Ad5 E1-TC and Ad5 E1A-TC so it is possible that this represents a unique effect of products from the Ad5 region. Presumably, increased expression of PPP1R1A would have a beneficial effect on virus survival by altering one of the many other pathways involving PP1; for example, the cell cycle. It is known that Ad E1A directly binds to pRb and releases the E2F transcription factor from pRb family-mediated repression resulting in reprogramming of cells to enter S phase [reviewed in [6]]. The increase in PPP1R1A could complement the Ad E1A mediated displacement of E2F from pRb by preventing PP1 activating (dephosphorylating) pRb; this would maintain cell cycle progression.

**Figure 12:**
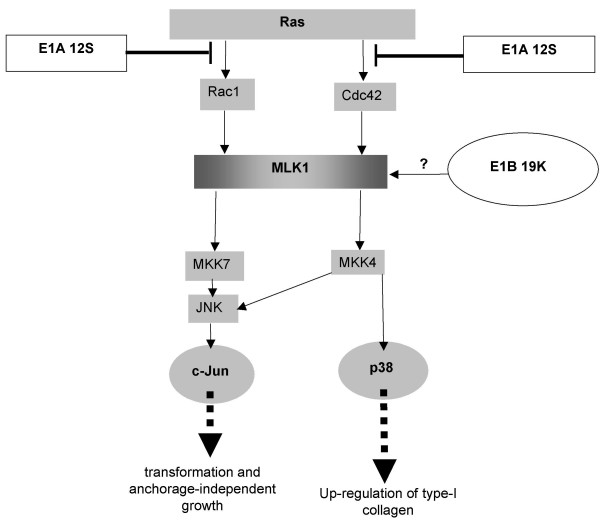
**Over-expressed levels of MLK1 could be causing preferential activation of the JNK pathway**. Rac1 or Cdc42 relieves MLK1 autoinhibiton and allows MLK1 to dimerise, autophosphorylate and activate the MKK7/JNK/c-Jun pathway, and possibly the MKK4/p38 and MKK4/JNK/c-Jun pathways. Wild-type E1A Ad 12S is known to inhibit Ras-mediated activation of Rac1 and Cdc42. The E1B 19K protein can activate the MKK7/JNK/c-Jun pathway; this could possibly be via MLK1.

The results also indicated that another possible unique effect of Ad5-transformation could be elevated expression of MLK1. Perhaps the over-expressed levels of MLK1 in Ad-transformed cells are causing preferential activation of the JNK pathway, as indicated in Figure 12, leading to transformation and anchorage-independent growth.

Levels of EGFR in Ad-transformed cells have already been well-characterised. Over-expression of EGFR has been observed in a variety of tumour types and it is thought that this may induce aberrant signalling, giving rise to uncontrolled cellular growth [reviewed in [61]]. Several lines of evidence suggest that the significant down-regulation of EGFR observed in Ad5 E1-TC makes sense both in the context of kidney cells and lack of oncogenicity of this Ad serotype: over-expression of EGFR is associated with poor prognosis and metastatic spreading of renal cancer [62,63] and monoclonal anti-human EGFR antibody has been found to increase delay in tumorigenesis of human kidney cancer cell lines [64].

The effect on the development process of tumour cells exerted by dysregulation of the other targets within these categories is less well-defined. Interference of human PAK4 mRNA by siRNA has been reported to decrease anchorage-independent growth of HeLa cells [65], suggesting that the observed down-regulation of PAK4 would adversely affect anchorage-independent growth. Human stathmin (STMN1) antisense DNA decreases the growth of K562 cells [66], so possibly up-regulation of STMN1 has a stimulatory effect on growth. The effect of up-regulation of MTA3 on the tumour cell line is even less clear: as it is known that the fate determination of H929 cells is dependent on MTA3 [67], perhaps MTA3 is playing a pivotal role in fate determination of the transformed BRK cells. However, the high variance associated with the duplicates for MTA3 suggests that the dysregulation may not be significant (Additional file 1).

A particularly well-characterised effect of dysregulation of host cell gene expression by Ad transformation of BRK cells is the down-regulation of surface expression of MHC Class I in Ad12-transformed rodent cells, which plays a major role in the evasion of the rodent immune response to Ad12-TC. It is known that the down-regulation of MHC class I molecules on the cell surface of Ad12 E1-transformed cells is due to decreased binding of NF-κB and increased binding of the repressor COUP-TFII to the enhancer, with the converse occurring in Ad5-transformed cells [reviewed in [6]]. However, this is thought to be due to the effect of Ad12 on the p50 component of NFkB (as opposed to RelA) as hypophosphorylation of p50 in Ad12-TC renders the NFκB heterodimer incapable of binding DNA [68], so is not likely to be affected by dysregulation of levels of RelA by Ad transformation. However, it is possible that the less-marked increase in RelA expression in Ad12 E1A-TC observed in this study could be acting to complement inhibition of the TNFR1 signalling pathway to prevent proapoptotic or proinflammatory signalling.

However, it is important to appreciate that down-regulation of MHC Class I alone does not govern oncogenesis. A suggestion of the complexity involved is provided by the fact that many genes that play roles in the stress and immune response are also implicated in other pathways within the cell: for example, NFAT5 and GADD45B.

The up-regulation of NFAT5 at the RNA level in Ad5-TC was confirmed by real-time PCR, however the real-time PCR results for the Ad12-TC were more variable (figure 11). The DNA binding domain of NFAT5 is thought to have evolved from NF-κB and, as NFAT5 can be induced by a wider range of stimuli than just the best characterised one of osmotic stress, the protein is thought to be a potential player in a wide range of cell signalling pathways; for example, studies in NFAT-5 deficient mice have suggested roles in embryonic development, integrin-induced cellular migration, and proliferation [69].

GADD45B, another member of the stress and immune response category, is up-regulated in normal cells in response to environmental stresses, mediates p38/JNK pathway activation by binding and activating MTK1/MEKK4 kinase and has been found to regulate Cdc2 and p21^WAF1 ^activities, resulting in G_1 _arrest, inhibited cell cycle progression, and apoptosis [70-72]. As down-regulation of GADD45B is associated with neoplasia [47], it is perhaps not surprising that GADD45B was found to be significantly up-regulated in BRK cells stably transformed with the non-oncogenic Ad5 E1 region (Additional file 1).

The other heavily populated GO categories contain genes related to cell cycle and proliferation and matrix, cell adhesion and the cytoskeleton: perhaps unsurprisingly, as these processes are known to be altered by the transformation process. The real-time PCR and immunofluorescence microscopy results confirmed the microarray study finding that significant down-regulation of collagen type I, alpha I occurs as a result of transformation of BRK cells with both Ad5 E1A and Ad12 E1A. Collagen type I is a major component of the extra cellular matrix (ECM) and the observed down-regulation is presumably involved in the loss of growth contact inhibition displayed by transformed cells.

**Figure 13:**
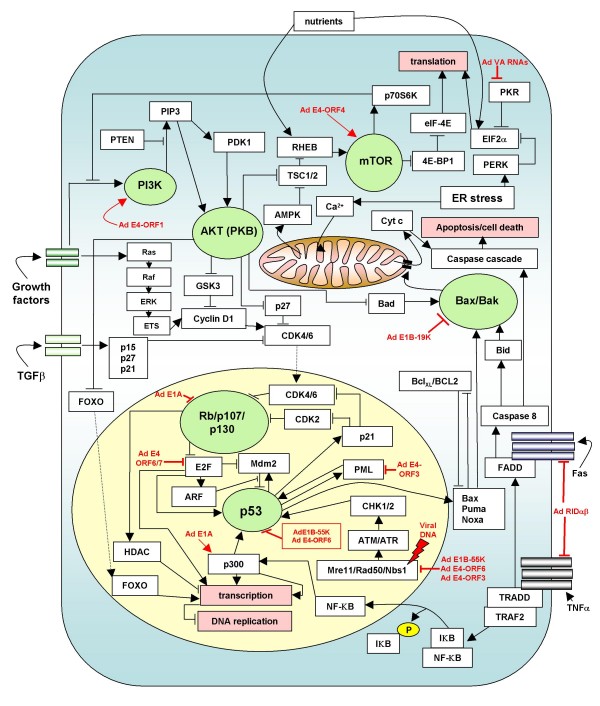
**Growth regulatory pathways of the host cell are perturbed by adenovirus**. Ad proteins act on the host cell signalling network to uncouple DNA replication from anti-growth signals, senescence and apoptosis. Similar growth deregulation is caused as a result of tumour cell mutations. Therefore, viruses and viral proteins represent a natural system that is used to study growth deregulation programs with the aim of gaining insight into the process of carcinogenesis. Figure adapted from [5].

The lesser populated GO categories defined in Figure 2 are no less important. For example, products of the Ad E1 region are known to be involved in subverting the host cell apoptotic response to viral infection. Only four genes related to apoptosis were found to be dysregulated by Ad transformation. One of these, ZMAT3 (rat synonyms PAG608 and WIG1), has been found to increase apoptosis [73] and decrease growth of tumour cells [74]. The ZMAT3 protein contains three zinc finger domains and a nuclear localization signal. Wildtype p53 up-regulates both mRNA and protein produced from the ZMAT3 gene. As over-expression of ZMAT3 inhibits tumour cell growth, a role for this gene in the p53-dependent growth regulatory pathway seems probable. Comparative analysis of all dysregulated genes across the three cell lines revealed the p53 canonical pathway to be significantly affected (Figure 4). This is unsurprising, given that p53 is central to numerous pathways already known to be disturbed by adenovirus transformation, as indicated in Figure 13.

Approximately 30% of identified genes had associated GO processes that did not readily fall into any of the categories mentioned in Figure 2 (a full listing of these transcripts is available in Additional file 1). The transcripts which fall into this multifaceted 'other' category are nevertheless important considerations in the conundrum of what governs adenovirus transformation. For example: tissue factor pathway inhibitor (TFPI) appears in two high scoring networks (Figure 7a; network 2 and Figure 10a; network 5) which share a degree of overlap with other statistically significant networks (Figures 7c and 10c). Cancer is one of the top biofunctions associated with these merged networks and two molecules dysregulated by Ad5 E1 transformation of BRK cells show a statistically significant association with this category (p-value ranging from 2.62 × 10^-9 ^to 2.8 × 10^-4^): TFPI and zinc finger matrin type 3 (ZMAT3). Up-regulation of TFPI mRNA is associated with human metastatic colorectal cancer [40]. TFPI regulates the proteolytic function of the TF-VIIa complex – a major initiator of coagulation pathways involved in wound healing *in vivo *[75]. Given that, pathologically, human solid tumours such as colon cancer are characterised by desmoplasia and tumour stroma has been compared to a 'wound that does not heal' [76], it is perhaps unsurprising that the interaction of TFPI and TF-VIIa has been implicated in tumour cell adhesion and migration [77].

## Conclusion

The classification of genes corresponding to RNA transcripts that were found to be up-regulated by microarray reported here demonstrates that many different processes have been altered as a result of Ad transformation. This underlines the difficulty in unravelling the cellular networks involved in the transformation process and oncogenesis. The largest group were classified as genes encoding for proteins involved in signal transduction. One of the hallmarks of neoplastic growth is the deregulation of cellular signalling pathways. It may be possible in the future to gain insight into the dysregulation at the protein level with advances in methods for producing oncoproteomic and oncogenomic data sets and bioinformatics. Our dataset serves as a starting point for further investigation into dysregulation of host cell networks as a result of adenovirus transformation. It is important to note that the relative low number of entries limits the comparative expression of genes dysregulated by Ad transformation presented in this paper. In addition, the 15,481 unique genes screened by the microarray filters used in this study represent 62% of the rat genome. As more is found out about the rat genome, it is conceivable that data relating to genes that are currently uncharacterised in this study, for example those that lack clear identities or are defined as ESTs, will yield more insights into the process of adenovirus transformation. Further work is required to investigate which splice variant of Ad E1A is responsible for the observed dysregulation at the pathway level and the mechanisms of E1A-mediated transcriptional regulation.

## Methods

### Cell culture and extracts

Primary BRK cells were isolated from 5 to 7 day old Wistar rats. The kidneys were removed aseptically, trypsinized, and cells isolated from the tissue samples were seeded out and harvested once sub-confluent [78]. Levels of mRNA expression in the primary BRK cells were compared to those in BRK cells transformed with the left-hand 16% of the viral genome containing the E1 region of Ad12 (Ad12 E1-TC; the RFC-1 cell line, as described in [79]) or Ad5 (Ad5 E1-TC; the Ad5 Xho cell line). Levels of mRNA expression were also examined by real-time PCR in clonal BRK cell lines transformed with Ad12 (Ad12-TC; the Ad12#1 cell line) or Ad5 (Ad5-TC; the Ad5#2 cell line) in which the infectivity of the input virus had been reduced by UV treatment. All four Ad-transformed rat cell lines contained integrated copies of the respective transforming (E1) region and expressed the transforming E1A and E1B proteins ((K. Raska, personal communication and GEB, unpublished results). In addition to the cell lines used in the real-time PCR analysis, the expression levels of targets at the protein level was also investigated using BRK cells immortalised with Ad12 E1A only (Ad12 E1A-TC; the AccIH cell line) and Ad5 E1A only (Ad5 E1A-TC; the HpaIE cell line [17]). Expression levels were also assessed in untransformed Fisher rat embryonic fibroblasts (3Y1 cell line). All transformed cell lines were maintained in high glucose Dulbecco's Modified Eagle Medium (DMEM) containing streptomycin (100 μg/ml), penicillin (100 I.U./ml), L-glutamine (2 mM) and 10% foetal calf serum (FCS; Invitrogen Ltd) in a humidified atmosphere of 5% CO_2 _at 37°C.

For microarray and real-time PCR analysis, total RNA was extracted from harvested primary BRK cells and transformed cell lines by the Trizol method and resuspended in RNase and DNase-free H_2_O. For Western blotting analysis, total protein was isolated from subconfluent cells harvested in ice-cold PBS, centrifuged at 1500 rpm for 5 min (Sorvall TC, Dupont) and resuspended in modified RIPA buffer [30 mM HEPES-KOH, 150 mM NaCl, 1% Triton-X100, 0.1% SDS, 1% sodium deoxycholate, 5 mM EDTA (pH7.6)] supplemented with protease inhibitors (Complete, Mini, EDTA-free protease inhibitor cocktail tablets, Roche) and phosphatase inhibitors (phosphatase inhibitor cocktails 1 and 2, Sigma) following the manufacturer's instructions. Cells were lysed for 1 hr on ice and centrifuged at 13000 rpm for 5 min at 4°C. Supernatant was assayed for protein concentration by the BioRad detergent compatible (DC) protein assay (BioRad) using a standard curve constructed from a dilution series of BSA in modified RIPA buffer.

### Antibodies

The following antibodies were used for Western Blotting (WB) or immunofluorescence microscopy: anti-Ad12 E1A mouse monoclonal antibody provided as undiluted culture medium (163; kind gift from Dr R. Grand), for Ad12 E1A WB; mouse monoclonal adenovirus type 5 antibody (M58; Abgent), for Ad5 E1A WB; anti-NF-κB (RelA) rabbit polyclonal antibody (Ab-1; Calbiochem), for RelA WB; purified rabbit polyclonal antibody (Abgent), for MLK1 WB; rabbit-anti-DARPP-32 affinity-purified antibody (Chemicon), for PPP1R1A WB; anti-collagen, type I rabbit polyclonal antibody provided as undiluted serum (Calbiochem), for detection of COL1A1 by immunofluorescence microscopy.

### Microarray experiments

Two different batches of the Rat GeneFilters^® ^microarray set comprising gf300, gf301 and gf302 (ResGen™, Invitrogen) were used in this study. Each genefilter comprises a nylon membrane spotted with cDNA clones that have been partially sequenced, verified against the GenBank database using BLAST and cross-referenced to the UniGene database. In order to minimize false positives arising from non-specific cross-hybridisation, the first 1,000 base pairs from the 3' untranslated region (UTR) are included in the cDNAs. In addition, each filter contains β-actin control spots and total genomic DNA 'landing lights' to enable correct orientation of each filter.

#### (i) Preparation of cDNA probe and microarray hybridization

Filters were pre-hybridised at 42°C for 2 h in 7.5 ml MicroHyb buffer (ResGen™) containing 7.5 mg of heat denatured mouse Cot-1 DNA (Invitrogen) and 7.5 mg GeneFilters primer poly dA. Each cell type was screened in duplicate using cDNA from the same preparation. Briefly, 10 μg of total RNA from primary BRK cells, Ad5-E1 and Ad12 E1 transformed BRK cells was reverse transcribed in the presence of oligo(dT)_12–18_, 1.5 μl of a 20 mM equimolar mix of dATP, dGTP and dTTP, and 10 μl [^33^P] dCTP (10 mCi/ml, specific activity 3000 Ci/mmol; ICN Radiochemicals). Following incubation with 1 μl of 0.1 M dithiothreitol, reaction buffer and 1.5 μl of Superscript II reverse transcriptase (Invitrogen) at 37°C for 1.5 h, probes were purified by chromatography on Bio-Spin-6 columns (BioRad) and hybridised to cDNA microarray membranes at 42°C for 16 h in MicroHyb solution (ResGen). Membranes were washed twice for 20 min in 2 × SSC-1% SDS at 50°C and once for 15 min in 0.5 × SSC-1% SDS at 55°C and exposed to a phosphoimager plate for 48 hr.

#### (ii) Data collection, normalization and analysis

The arrays were scanned using a Molecular Dynamics Storm Phosphoimager at a resolution of 50 μm. The data were quantified by using the Pathways v.2.01 software (Research Genetics). The data were normalised using the average expression intensity of all of the spots on the filter which gave results consistently centred on the ideal gene expression ratio of 1, indicating no change between duplicate samples. The data normalised with respect to all data points was used in subsequent analysis and expression levels below twice the background level were excluded. Based on assessment of the relative sensitivity of the GeneFilters arrays and the average experimental error, an expression ratio of 1.5 was chosen to define genes that are differentially expressed. The data discussed in this publication have been deposited at the EBI ArrayExpress website and are accessible under the accession number E-MTAB-30, in accordance with MIAME standards.

The rat genome has been sequenced since the construction of the arrays therefore bioinformatics analysis was carried out to validate the results from the microarray analyses. The accession numbers of cDNA clones from the microarray data that corresponded to differentially regulated genes were subjected to BLASTN searches against the expressed sequence tag (EST) database. Returned EST gi numbers were searched against BLASTN nr database to identify corresponding mRNA record containing protein and gene identity.

Initial information on pathways and gene ontology (GO; [80]) functions of genes and proteins were obtained from the database for annotation, visualisation and discovery; DAVID [81], the Gene Map Annotator and Profiler; GenMAPP [82], and the Kyoto Encyclopaedia of Genes and Genomes (KEGG) databases integrated at GenomeNet Database Service .

The Ingenuity Pathways Analysis (Ingenuity Systems^®^, ) applications were used to generate networks and assess statistically relevant biofunctions and canonical pathways associated with the microarray data. A data set containing the accession numbers of microarray clones and corresponding expression values was uploaded and mapped to corresponding genes in the Ingenuity knowledge base to enable the construction of networks and analysis of biofunctions and canonical pathways. A fold change cut-off of 1.5 was set to identify genes whose expression was significantly differentially regulated. These genes, called focus genes, were overlaid onto a global molecular network developed from information contained in the Ingenuity knowledge base. Networks of these focus genes were then algorithmically generated, based on their connectivity.

The BioFunctional analysis identified the biological diseases that were most significant to the data set as a whole (comparative analysis) or to the network. Genes from the dataset (or network genes) that met the fold change cut-off of 1.5 and were associated with biological diseases in the Ingenuity knowledge base were considered for the analysis. Fischer's exact test was used to calculate a p-value determining the probability that each biological disease assigned to that data set (or network) is due to chance alone. The Functional Analysis of a network identified the biological diseases that were most significant to the genes in the network.

Canonical Pathways Analysis identified the pathways from the Ingenuity Pathways Analysis library of canonical pathways that were most significant to the dataset. Genes from the dataset that met the fold change cut-off of 1.5 and were associated with a canonical pathway in the Ingenuity knowledge base were considered for the analysis. The significance of the association between the dataset and the canonical pathway was measured in two ways: firstly, a ratio of the number of genes from the dataset that met the expression value cut-off that map to the pathway divided by the total number of molecules that exist in the canonical pathway is displayed. Secondly, Fischer's exact test was used to calculate a p-value determining the probability that the association between the genes in the dataset and the canonical pathway is explained by chance alone.

### Quantitative real-time PCR

#### (i) Preparation of gene-specific PCR primers

Gene-specific primer pairs were designed using PrimerExpress version 1.5 software (Applied Biosystems) and purchased from Eurogentec. The uniqueness of the designed primer pairs was checked by a BLAST homology search  to ensure that homologous genes were not cross-amplified by the same primer pair. Where possible, primers were designed that span intron/exon boundaries, and a list of all primers for target genes examined in this study is given in Table 1. Rat glyceraldehyde-3-phosphate dehydrogenase (GAPDH) was used as a housekeeping mRNA and the sequences for the forward and reverse primers (RTPrimerDB identification number 192) were obtained from the Real Time PCR Primer and Probe Database .

#### (ii) RNA preparation and first-strand cDNA synthesis

Following total RNA extraction, the RNA solution was treated with RQ1 RNase-free DNase (Promega) and RnaseOUT Recombinant Ribonuclease Inhibitor (Invitrogen), following the manufacturer's instructions. Total RNA samples were analysed by gel electrophoresis for the presence of 28S and 18S rRNA. Purity and concentration of RNA samples was determined by UV spectroscopy [as described in 83]. First-strand cDNA synthesis (1 μg total RNA per 20 μl reaction) was carried out with the ImProm-II reverse transcription system (Promega) using an oligo(dT) primer (Amersham Biosciences), following the manufacturer's instructions. Reactions containing all reagents except reverse transcriptase were performed for each total RNA sample to test for the presence of contaminating DNA.

#### (iii) Real-time PCR

PCR amplification was carried out in 96-well plates with adhesive covers using the ABI Prism 7900 HT instrument (Applied Biosystems) for all targets except NFAT-5 which was amplified using the GeneAmp 5700 thermocycler. The efficiencies of target gene and GAPDH amplification were investigated by performing a dilution series of total RNA (1 μg to 0.1 ng) following recommendations [84]. Each 25 μl reaction contained: 12.5 μl 2 × SYBR Green PCR master mix (Applied Biosystems), 1 μl forward primer (10 μM), 1 μl reverse primer (10 μM) and 1 μl of cDNA. Each sample was analysed in triplicate. To test for the presence of contaminating DNA, 25 μl reactions containing 1 μl RNAse DNase-free H_2_O in the place of either 1 μl reverse transcriptase or cDNA were analysed alongside reactions containing reverse transcriptase and cDNA for each sample. Each real-time PCR program was 2 min at 50°C, 10 min at 95°C, followed by 40 repeats of 15 s at 95°C and 1 min at 60°C. A final dissociation stage added to each run (15 min at 95°C, 20 s at 60°C and 15 s at 95°C) enabled assessment of presence of contamination, primer-dimers or non-specific PCR products. Some PCR products were also assessed by agarose gel electrophoresis.

#### (iv) Data analysis

Data was analysed by the 2^-ΔΔC^T method [85]. Any raw C_T _values within the repeats that differed by a standard deviation of > 0.3 were not included in the calculation of average C_T _[84]. The target transcript threshold cycle (C_T_) values obtained from the SDS 2.2 software (Applied Biosystems) were normalised for GAPDH C_T _values to give ΔC_T_. The comparative CT method was used to analyse the real-time PCR data for targets with equal amplification. In brief, the target transcript ΔC_T _values for the transformed cells were expressed relative to the ΔC_T _values of the primary BRK cells to give ΔΔC_T_, and the fold change in expression relative to the primary BRK cells was calculated by 2^-(ΔΔCT)^. Real-time PCR data obtained for NFAT5 amplification from the GeneAmp 5700 SDS software was assessed by the standard curve method. The statistical significance of the raw C_T _values representing differences in mRNA expression levels between BRK cells and transformed cells was determined by two-tailed student's *t*-test assuming equal variances.

### Western blot analysis

Isolated cell extract protein (Ad12 E1A WB: 60 μg/lane, Ad5 E1A WB: 20 μg/lane, RelA WB: 20 μg/lane, MLK1 WB: 25 μg/lane, PPP1R1A WB 50 μg/lane) was diluted in red loading buffer (NEB) containing 125 mM DTT (NEB), heated at 100°C for 5 min and then applied to a 5% polyacrylamide stacking gel cast on top of resolving gel containing a percentage of polyacrylamide dependent on the molecular weight of target protein: Ad E1A and RelA, 10%; MLK1, 7.5%; PPP1R1A, 12.5%. After electrophoresis, the proteins were wet transferred onto either a polyvinylidene difluoride membrane (Millipore, UK) for AdE1A, RelA and MLK1 WBs, or nitrocellulose membrane for PPP1R1A WBs, using a BioRad mini-gel block. The membranes were treated overnight at 4°C with blocking solution (Ad E1A and RelA WBs: 0.1% Tween-phosphate-buffered saline [PBS-T] supplemented with 10% milk powder, PPP1R1A WB: PBS-T supplemented with 5% BSA, MLK1 WB: PBS-T supplemented with 3% BSA). The membranes were washed in PBS-T. Samples were incubated for 2 hr at room temperature with the primary antibody at an appropriate dilution (anti-Ad12 E1A, 1:100; anti-Ad5 E1A, 1:400; anti-RelA, 1:5000; anti-MLK1, 1:500; anti-PPP1R1A, 1:1000) in PBS-T supplemented with either 5% milk powder for Ad E1A and RelA WBs or 3% BSA for MLK1 and PPP1R1A WBs. The membranes were washed for 30 min at room temperature with three changes of PBS-T. Incubation with the secondary antibody conjugated to horseradish peroxidase (Sigma) (anti-mouse HRP for Ad E1A WBs; anti-rabbit-HRP for MLK1, PPP1R1A and RelA WBs) was for 1 hr at room temperature, at appropriate dilutions (Ad E1A WBs, 1:1000; MLK1 WB, 1:50 000; PPP1R1A WB, 1:5000; RelA WB, 1:1000) in same diluents as were used for primary antibodies. Membranes were washed again for 30 min, imaged, washed again for 30 min in PBS-T then incubated for 2 hr at room temperature with an anti-GAPDH antibody (Calbiochem) diluted 1:20000 in PBS-T supplemented with 5% milk powder. Incubation with the secondary antibody (anti-mouse-HRP, Sigma), diluted 1:2000 in PBS-T supplemented with 5% milk powder, was for 1 hr at room temperature. To detect HRP activity, advanced or normal ECL reagents (Amersham) were used on membranes following initial staining for target protein or subsequent staining for GAPDH, respectively. Images were captured with a Fuji LAS-3000 CCD camera (Fuji, Tokyo, Japan) and AIDA image analysis software was used to determine band intensities. The band intensities for target proteins were corrected for background and normalised using GAPDH expression calculated for the same lane.

### Immunofluorescence microscopy

Cells were cultured in six-well plates containing sterile coverslips until they were approximately 70% confluent, washed twice with PBS and incubated with Sigma Fix (1 ml/well) at room temperature for 5 min. Cells were washed twice with PBS and incubated at room temperature with 1% Triton X-100 in PBS (1 ml/well) for 10 min. Cells were washed once with PBS and incubated at room temperature for 10 min with 10 mM ammonium bicarbonate in PBS (1 ml/well). Cells were washed twice with PBS and blocked with 10% NGS in PBS (PBS_ngs_) at 37°C for 1 hr. Samples were incubated for 1 hr at room temperature with gentle shaking with the primary anti-collagen antibody, diluted 1:60 in PBS_ngs_. The cells were washed several times with PBS and secondary antibody was pipetted carefully onto each coverslip. Following 30 min incubation at room temperature in the dark with anti-rabbit TRITC diluted 1:100 in PBS_ngs_, coverslips were washed several times with PBS with DAPI in the final wash and transferred onto slides sample side down. Coverslips were mounted with a drop of mounting medium (Vectashield Hardset; Vector Laboratories Inc, USA) and edges were sealed with clear nail varnish. Cells were viewed with an Axioplan 2 microscope (Carl Zeiss Ltd, UK) using phase contrast and UV epifluorescence techniques. Captured images were analysed using AxioSet software (Carl Zeiss Ltd, UK) to calculate the mean intensities of the Texas Red and DAPI signals from ten separate fields taken from each slide. The mean intensity signal for collagen was corrected for background and normalised using DAPI intensity from the same field.

## Abbreviations

Ad: adenovirus; BRK: baby rat kidney; GO: Gene Ontology; IPA: Ingenuity Pathway Analysis; MHC: major histocompatibility complex; Rb: retinoblastoma.

## Authors' contributions

JS performed data analysis, validated targets by PCR and Western blot analysis and helped to write the paper; LJG performed microarray experiments and data analysis; PK performed data analysis and assisted with the microarray experiments; GEB conceived and designed the study and helped to write the paper.

## Supplementary Material

Additional file 1 **Transcripts dysregulated by adenovirus E1 transformation.** Summary of transcripts that were differentially expressed as determined by the DNA microarray technique, using criteria outlined in the Methods section and presented in three tables listing (1) the named genes with gene ontology annotation, (2) the genes identified by bioinformatic analysis of dysregulated transcripts but lacking gene ontology annotation, and (3) transcripts that lack gene identity and annotation. Click here for file
